# 

**Published:** 1829

**Authors:** 


### CONTENTS OF NO. I, VOL. III.

### ESSAYS AND CASES.

ART. I.—Practical Essays on Medical Education and the Medical Profession in the United States.—BY THE EDITOR. - 13

*Essay I.—Selection and Preparatory Education of Pupils.*

II. An account of the Bilious Remittent Fever of 1828; as it prevailed in Circleville, and its vicinity; and, especially, along the line of the Ohio and Erie canal, near that town. By DR. JOHN COOK BENNETT. - - - - 27

III. Notes of case of Gutta Serena of the right eye, from the pressure of a tumour on the optic nerve, By JEDEDIAH COBB, M. D. Professor of Anatomy, in the Medical College of Ohio. 33

IV. Diagnostic notices of a case of Fungus Hæmatodes; designed as a contribution to the history of that disease. By the EDITOR. - - - - - 34

V. History of a case of Verminous Disease. By BENJAMIN S. BROWN, M. D. of Logan County, Ohio. - - 40

VI. MEDICAL JURISPRUDENCE.—Report of a trial for murder, in which the culprit was defended on the ground of his labouring under Mania a potu, or Delirium from Intemperance. By the EDITOR. - - - - - 44

VII.—History of a case of Empyema from protracted Measles and Pleurisy, in which the operation of Paracentesis gave immediate relief, By Dr. SAMUEL MERRIWETHER, of Jeffersonville, Ind. - - - - - - 65

### REVIEWS AND BIBLIOGRAPHICAL NOTICES.

ART. VIII.—A treatise on the Diseases of the Chest, in which they are described according to their anatomical characters; and their Diagnosis is established on a new principle by means of Acoustick instruments, with plates. Translated from the French of R. T. H. Laennec, M. D. with a preface and notes By JOHN FORBES, M. D.

A short treatise on the different methods of investigating the diseases of the Chest. Translated from the French of M. Collin. By W. N. RYLAND, M. D. From the third London edition; with plates and an explanatory introduction by a fellow of the Massachusetts Medical Society. - - 68

IX. A Manual of Practical Obstetrics: arranged so as to afford a concise and accurate description of the Management of preternatural Labours; preceded by an account of the Mechanism of Natural labour. From the French of Julius Hatin, Doctor of Medicine of the Faculty of Paris, Professor of Obstetrics and of the diseases of Women and Children, &c. &c. By S. D. Gross, M. D. 100

X. Transactions of the Medical Society of the State of New-York, for the year 1829, with the annual Address by T. Romeyn Beck, M. D. President of the Society, 1829. 104

XI. An Act to incorporate Medical Societies for the purpose of regulating the Practice of Physic and Surgery in Ohio. Together with the proceedings of the General Medical Society. Columbus: 1829. - - - - - 107

---

### MISCELLANEOUS INTELLIGENCE.

### I. Analectic.

1. Amputation of the neck of the Uterus. - - 109
2. On the treatment of Cancerous Affections of the Cervix Uteri, and especially on amputation of that part, by M. Lisfranc; reported by M. Avenil. - - - 110
3. Treatment of Varicose Ulcers. - - 114
4. Lithontripteur. - - - 117
5. Action of Colchicum on the Urine. - 118
6. New mode of treating Tænia, discovered by Dr. Schmidt, of Berlin, and described by M. Caspar, by order of the Prussian Government. - - - - 118
7. Neuralgia Facialis. - - - 120
8. Dysentery. - - - 121
9. Complete Amaurosis cured by the application of Leeches to the Nasal Fossæ. - - - 122
10. Remarkable superiority of amputating by one incision through the Integuments and Muscles. - 122
11. On Blistering Infants. - - - 123
12. Neuralgia. - - - - 123
13. Ascites cured by Pressure. - - 123
14. Treatment of Strictures of the Urethra. - 124
15. Mode of Preserving Specimens of Morbid Anatomy. - 125
16. Danger of Artificial Inflation of the Lungs. - 125
17. Iodine for Chilblains. - - - 126
18. Sting of a Wasp. - - - 126
19. Success of cold effusions in Hysteria. - - 126
20. Radical cure of a Hydrocele, by the introduction of a needle into the Tunica Vaginalis. - - 126
21. Nux Vomica in Chronic Diarrhæa and Intestinal Hemorrhage. - - - - - 127

22. Diagnostic Symptoms of Dislocation of the Head of the Femur into the Ischiatic Notch. By Dr. BUCHANAN. - 127
23. Traumatic Tetanus—Pathology of. - - 129
24. Ointment for Glandular Swellings. - - 130
25. Caustic Paste. - - - 130
26. Collyrium Siccum for the Opacity of the Cornea. - 131
27. The Effects of entire Abstinence from Solid and Liquid Food. 131
28. Of the Medicinal properties of the Leontodon Taraxacum. 134

II. ANALYTICAL.

29. Mania a potu. - - - 135
30. Neuralgias. - - - - 138
31. Colica Pictonum. - - - 140
32. Flour a remedy for Burns and Scalds. - - 141
33. Fractures of the os Femoris. - - 142
34. Test for Prussic Acid. - - - 143
35. Depletion a cure for Gangrena Senilis. - 144
36. Angina Pectoris. Nervous System. - - 146
37. Metallic Ligatures in Surgery. - - - 148
38. Treatment of the Cutaneous disease produced by certain vegetable poisons. - - - - 148
39. The London University. - - - 149
40. Fatal Illness and Dissection of Dr. Gall. - 151
41. Death of Professor Gorham. - - 153
42. Death of a Centenarian. - - 154

III. ORIGINAL.

43. Small Pox. - - - - 155
44. Fatal Effect from Vaccination. - - 156
45. Extensive cellular inflammation from Vaccination. - 158
46. Bath Chalyeate Spring. - . - 159
47. First District Medical Society of Ohio. - - 160

